# Assessment of soluble immune mediators as potential biomarkers during immune checkpoint inhibitor therapy

**DOI:** 10.18632/oncotarget.26749

**Published:** 2019-03-08

**Authors:** Norikazu Matsuo, Koichi Azuma, Tetsuro Sasada

**Affiliations:** Division of Respirology, Neurology, and Rheumatology, Department of Internal Medicine, Kurume University School of Medicine, Kurume, Fukuoka, Japan; Cancer Vaccine Center, Kanagawa Cancer Center Research Institute, Yokohama, Kanagawa, Japan

**Keywords:** PD-1, immune checkpoint inhibitor, biomarker, CXCL2, MMP2

In recent years, the treatment strategy for advanced or recurrent non-small lung cancer (NSCLC) has changed drastically with the development of immune checkpoint inhibitors (ICIs). Nivolumab and pembrolizumab, ICIs that target programmed death (PD)-1, restore the immune system's capacity to recognize and eliminate tumors, thus improving treatment outcome in NSCLC patients. Nevertheless, since it has been reported that only a limited number of NSCLC patients show marked and durable responses to anti-PD-1 therapy, there has been a need for biomarkers that can predict whether anti-PD-1 therapy would be clinically beneficial [1].

Since anti-PD-1 therapy targets the immune system, biomarkers associated with immune responses must be developed. For example, the level of PD-L1 expression on tumor cells, as assessed by immunohistochemistry (IHC), has already been highlighted as a potential biomarker predictive of the response to PD-1 inhibitor. However, the reliability of PD-L1 expression as such a marker appears to be limited because it can be quite heterogeneous, even within the same tumor, and may change dynamically and drastically according to circumstances [1]. In addition, biopsy of tumors to assess IHC-based PD-L1 expression requires invasive procedures such as bronchoscopy or video-assisted thoracoscopy, and can sometimes be problematic depending on the size and location of the investigated tumors. By contrast, markers present in blood can be assessed with a minimal degree of invasiveness, and unlike biopsies, blood samples can be repeated and sequentially studied, thus providing dynamic information during treatment. These advantages of blood sampling would be well suited to patients receiving ICIs, in view of the dynamic nature of the antitumor immune response.

Recently, several studies have suggested that changes in serum soluble factors are associated with the outcome of anti-PD-1 therapy. Wu et al. have reported that patients with metastatic melanoma receiving nivolumab or pembrolizumab showed a poor response and overall survival when they displayed an increase in serum angiopoietin-2 [2]. Fujimura et al. have also suggested that an increase in the serum level of soluble CD163 was associated with a better response to nivolumab in patients with advanced cutaneous melanoma [3]. On the other hand, a decrease in the level of serum IL-8 was significantly associated with a better response and longer overall survival in patients with NSCLC and melanoma receiving nivolumab or pembrolizumab [4]. These findings support the possibility that assessment of early immune responses can predict the outcome of anti-PD-1 therapy, and that monitoring the levels of soluble immune mediators in peripheral blood might be a new strategy for investigation of treatment-related biomarkers.

Recently we have adopted this approach, and have assessed the levels of 88 different soluble immune mediators in peripheral blood of NSCLC patients before and after anti-PD-1 treatment in relation to clinical outcome [5]. Although soluble immune mediators in pretreatment samples were not associated with the outcome of anti-PD-1 therapy, decreasing levels of CXCL2 and increasing levels of MMP2 after treatment were significantly correlated with longer progression-free survival. CXCL2 is a chemokine that plays a critical role in the chemotactic recruitment of neutrophils [6]. Additionally, CXCL2 promotes recruitment of myeloid-derived suppressor cells (MDSCs)—critical contributors to suppression of the immune response [7]—to the tumor bed *via* signaling mediated by CXCR2 (the receptor for CXCL2) [8]. Our results suggest that the post-treatment decrease in the level of CXCL2 might prevent CXCR2- mediated accumulation of MDSC in the tumor bed, thus enhancing the efficacy of PD-1 checkpoint blockade. Furthermore, MMP2 has been found to play a role in degradation of the extracellular matrix [9]. It is possible that an increase in the level of MMP2 after anti-PD-1 therapy might promote lymphocyte accumulation in the tumor microenvironment and an immune-mediated anticancer response. However, details of the mechanistic role of MMP2 are still unclear. Additional prospective studies of the complex biology influencing patient responses to treatment are warranted.

Sequential assessment of CXCL2 and MMP2 levels in peripheral blood was also shown to be helpful for response monitoring in NSCLC patients. Decreasing and increasing levels of CXCL2 and MMP2, respectively, were maintained during the course of anti-PD-1 therapy in patients who showed better clinical outcomes, even in those with tumor pseudoprogression. Additionally, disease progression coincided with plasma CXCL2 elevation above the baseline [5]. The dynamic changes in these factors might reflect the antitumor immune response. Interestingly, a recent study using mouse models has demonstrated that the anti-tumor effect of anti-PD-1 was significantly enhanced when accumulation of MDSC in the tumor bed was inhibited by CXCR2 deficiency or anti-CXCR2 monoclonal antibody therapy [10]. Taken together, the data suggest that CXCL2 has great potential as a biomarker and therapeutic target for anti-PD-1 therapy.

Although anti-PD-1 therapy has promising effects, resistance develops in most patients. Furthermore, there is still no standard monitoring strategy for detection of progression after such therapy. We have shown that changes in the plasma levels of CXCL2 and MMP2 are significantly associated with the clinical outcome of anti- PD-1 therapy. Since these soluble immune mediators in plasma can be easily measured by minimally invasive blood sampling, they could be useful for monitoring clinical outcomes in NSCLC patients receiving PD-1 inhibitor therapy.

## References

[1] Nishino M (2017). Nat Rev Clin Oncol.

[2] Wu X (2017). Cancer Immunol Res.

[3] Fujimura T (2018). Front Oncol.

[4] Sanmamed MF (2017). Ann Oncol.

[5] Matsuo N (2019). Int J Cancer.

[6] Guo C (2013). Vasc Cell.

[7] Balkwill FR (2012). J Cell Sci.

[8] Zhang H (2017). Oncogene.

[9] Choi JW (2017). Theranostics.

[10] Highfill SL (2014). Sci Transl Med.

